# Is something out of reach more attractive? The effectiveness of visual distance in computational advertising

**DOI:** 10.3389/fpsyg.2022.994573

**Published:** 2022-09-08

**Authors:** Tong Liu, Zhengdong Yu

**Affiliations:** School of Economics and Management, Wuhan University, Wuhan, China

**Keywords:** visual distance, computational advertising, CTR, CVR, construal level theory

## Abstract

With the development of mobile Internet technology, firms need to complete the entire process of consumer targeting, ad content generation, and ad display in a very short time window. Therefore, computational advertising, such as native ads on social media platforms, has become the mainstream of online advertising with its automation and personalization features. However, computational advertising faces some problems when using artificial intelligence technology to generate content. First, the images should have a significant enough impact on consumers and be easy to adjust to save computational power at the same time; second, the iteration of the computational advertising system relies on consumer behaviors or advertising effectiveness, and firms need to learn the relationship between ad design and consumer behaviors. Under the above two problems, this paper selects visual distance as the main variable, and images can be adjusted by cropping to save computational power. This paper incorporates image design and ad effectiveness metrics into the construal level theory framework, under which the effectiveness metrics can be quickly determined. Following previous studies, we use click-through rate (CTR) to represent the early stage of the sales funnel and a higher construal level and CVR (conversion rate) to represent the later stage of the sales funnel and a lower construal level. Therefore, visually distant images bring distant psychological distance or higher construal level, which can get higher CTR; visually proximate images bring near psychological distance or lower construal level, which can bring higher CVR. These findings suggest that firms can improve the efficiency of their advertising systems and gain more revenue by understanding consumer psychological states.

## Introduction

As mobile technology evolves, online and offline consumption scenarios continue to converge, and the window of time in which companies can display online advertising becomes shorter and shorter. Computational advertising, which relies on data and algorithms to drive it, has become the mainstream of Internet advertising, relying on its automation and personalization(Choi et al., 2020). Computational advertising is defined as a general, data-driven advertising approach that relies on an enterprise’s enhanced computing power, mathematical models/algorithms, and technological infrastructure to monitor individual behavior and create and deliver advertising messages (Huh and Malthouse, 2020). Feed ads and banner ads on social media platforms, for example, are typical examples of computational advertising that are presented differently, but each placement or display is done by a computational advertising system through a fixed paradigm (Sayedi, 2018). Technically, computational advertising selects target consumers by filtering consumer tags, uses AI-generated ad content and makes personalized adjustments, uses quantifiable metrics such as click-through rate (CTR) or conversion rate (CVR) to measure the effectiveness, and finally finishes adjusting and iterating on model parameters by collecting effectiveness data to complete more accurate display in the next cycle (Wang et al., 2019; Yun et al., 2020). Computational advertising relies on data and algorithms and accomplishes the goal of accurate delivery by mining data correlation. However, as consumers are exposed to a large amount of complex information on different media platforms and consumption scenarios everyday, it is increasingly difficult for companies to use an automated system to capture consumers’ attention and persuade them to buy(Ordenes et al., 2018; van Noort et al., 2020). On the one hand, advertisers use historical consumer behavior data to predict consumer demand and generate ad content based on that data, making the ads more personalized to meet their needs. However, it is difficult for the system to adjust the parameters in time, and the system can only keep testing and trial and error, which wastes a lot of advertising resources without achieving satisfactory results (Lee et al., 2020; Marchand and Marx, 2020). On the other hand, the time window for displaying ads is very short, and firms need to generate and adjust ad contents quickly. For example, RTB needs to be completed within 0.05 s, so the time-consuming and exquisite content creation method for print ads cannot apply to computational advertising (Sayedi, 2018). Therefore, computational advertising must use artificial intelligence to complete the creation of ads, and the ads should be able to use as little computation power as possible to adjust quickly and effectively while meeting personalized needs (Qin and Jiang, 2019; Choi et al., 2020).

The visual distance, or the image frame, refers to whether the image appears to be concrete and near, or abstract and far (Trope and Liberman, 2010; Kim et al., 2019b). In terms of computation power, the advertising system can accomplish the adjustment of visual distance by cropping the images. However, in terms of advertising effectiveness, previous studies have pointed out that the physical distance between elements in images and consumers can influence consumers’ attitudes (Jia et al., 2017) and that visual distance of images can also influence advertising attitudes, product evaluation, and purchase intention in print ads (Kim et al., 2019b). In the context of computational advertising image generation, the visual distance was chosen as the main variable in this study and was included in the construal level theoretical framework for analysis. In summary, automated computational advertising systems still need to be improved in terms of ad content design, they need to introduce guiding principles or frameworks for content generation and adjustment, and to complete iterations in conjunction with ad effectiveness metrics. Under the above two requirements, this paper constructs a corresponding guiding framework by introducing construal level theory into the automated system to complete the creation and delivery of ad content and improve advertising efficiency. When combined with computational advertising effectiveness metrics, the differences in the effectiveness brought by different images can be verified.

In terms of advertising effectiveness metrics, CTR and CVR, which are commonly used in computational advertising studies, are selected as dependent variables in this study (Wang et al., 2019; Yun et al., 2020). On the online advertising market side, CTR and CVR have different meanings for different players in this advertising market. Media platforms value CTR more because it represents the attractiveness of ads, and media platforms can price Internet traffic by CTR; advertisers value CVR more because it represents the actual purchase of consumers and can bring advertisers revenue (Choi et al., 2020; Yun et al., 2020). On the consumer side, clicks and conversions represent different stages of the consumer’s decision journey. The sales funnel or consumer decision journey is the process that begins with awareness, initial consideration, evaluation, purchase, and post-purchase behavior (Kietzmann et al., 2018). This study divides the decision journey into three stages, consistent with previous studies: pre-purchase, purchase, and post-purchase (Lemon and Verhoef, 2016). Specifically, consumers see the ad, to become interested and click on the ad belongs to the pre-purchase evaluation stage, where consumer information is processed in a more abstract and scattered way and is recorded as a click, so the CTR can reflect the attractiveness of the ad; after completing the evaluation, consumers will seriously consider whether to purchase or convert, where advertising information is processed in a more specific and focused way, and the corresponding purchase or download is recorded as a conversion, the CVR reflects the persuasiveness of the ad (Wang et al., 2019). Consumers can finish a decision journey quickly, and the goal of computational advertising is to increase the fluency of consumer information processing through a personalized ad and shorten the journey to achieve advertising effectiveness, so the matching of content and consumer journey stages is particularly important for computational advertising (Mafael et al., 2021). Therefore, this paper incorporates both ad content and ad effectiveness in the framework of construal level theory, which can enhance advertising efficiency. Specifically, visually distant images can evoke a higher construal level of consumers and match the pre-purchase stage to obtain a higher CTR; visually proximate images can evoke a lower construal level of consumers and match the purchase stage to obtain a higher CVR.

The data used in this study came from a large game company in China, which manages different types of games. We use the advertising campaign data of one new game. The company generated 116 advertising images for the game and launched them on Weibo, a well-known social media platform. Based on this unique data, this study systematically evaluates the impact of advertising images on effectiveness metrics. This study use the Heckman selection model to correct the self-selection bias caused by the advertiser (Heckman, 1979; Sande and Ghosh, 2018), and the results were validated using multiple models. The construal level theory was applied to correlate advertising content with consumer psychological states to enhance the efficiency of advertising. The results verified the usefulness of psychological theory in computational advertising.

## Theoretical background

### Advertising images: proximate vs. distant

On social media platforms, images can significantly influence consumers’ cognitive states (e.g., attention, attitudes, etc.; Kim et al., 2019a; Sevilla and Meyer, 2020) and behaviors (e.g., clicks, purchases, etc.; Young et al., 2019; Cian et al., 2020). Advertising images can improve advertising effectiveness by gaining consumers’ attention; for example, well-chosen images can improve ad attitudes and evaluations (Bruce et al., 2017). Existing studies suggest that the design of advertising images can influence advertising effectiveness, and the design can be divided into two dimensions. First, images have some inherent characteristics such as size and texture that can be considered as part of the image (Pieters et al., 2010). For example, the quality of the image (Hagtvedt and Patrick, 2008), the color (Pieters et al., 2010), and the arrangement of elements (Farace et al., 2020) have been shown to influence the viewer’s evaluation of an ad. Second, the elements presented in the images have expressive and narrative effects, such as the visual distance used in this study. For example, human and view selection in images can also improve consumers’ attitudes and purchase intentions (Guido et al., 2019; Northey et al., 2020). These expressive elements in images can influence advertising effectiveness by changing consumers’ perceptions of social distance or psychological closeness and processing fluency (Park and Morton, 2015; Roose et al., 2019).

The impact of advertising images on consumers in the computational advertising context should also be verified again. First, unlike well-designed images in print ads, images used in computational advertising on social media platforms come from multiple sources, and their resolution and aesthetics cannot be guaranteed (Zheng et al., 2019; Li and Xie, 2020). Moreover, computational advertising images require personalized adjustment according to consumer tags, and uniform images cannot meet the personalization requirements (Choi et al., 2020; Huh and Malthouse, 2020). In addition, the adjustment of ad images should also save computation power while meeting the format requirements of the platform for real-time bidding and delivery (Qin and Jiang, 2019; Choi et al., 2020). In this study, we choose the visual distance of images as the independent variable to meet the above requirements. The visual distance of an image belongs to the expressive features, which refers to the perception of the proximity of the content element in the image, i.e., whether the content in the image is represented in a focused manner and whether it presents a near or far feeling (Kim et al., 2019a). In the delivery of ads, the visual distance can be adjusted by cropping and enlarging the complete image, which not only requires less computation power but also can be effectively personalized.

In addition to the advantage of saving computation power, the advertising system can also adjust the visual distance of ad images to influence the way consumer’s process information and subsequent consumption behavior. This study uses the construal level theory to explain how visual distance affects consumer perceptions, attitudes, and behaviors. Increases in temporal, spatial, sensory, and social distance can increase the level of abstraction of consumers’ mental representations when making decisions (Trope et al., 2007; Trope and Liberman, 2010). High construal levels represent relatively abstract, context-independent representations of primary features; low construal levels represent relatively concrete, context-related representations of secondary features. Consumers can perceive spatial distances as distant when ads use visually distant images; they can perceive spatial distances as reduced when ads use visually proximate images. In previous studies, it is mainly the spatial distance of consumers from the image or the size of the image that is relevant (Jia et al., 2017; Kim et al., 2019a). In the context of computational advertising, it is difficult for firms to manipulate the spatial distance between images and consumers, as well as to adjust the size of images. Therefore, manipulating consumers’ information processing and subsequent behavior by adjusting the visual distance of images is a good option.

The link between the visual distance of an image and the construal level has also been pointed out in previous studies (Kim et al., 2019a; Roose et al., 2019), but this study differs in two ways. First, the advertising context is different; instead of media contexts in computational advertising, the previous study used print ads and laboratory experiments to test the hypothesis, emphasizing the effect of advertising attractiveness when it is consistent with the product type. This study, on the other hand, examines computational advertising on social media platforms, where consumers face more complex scenarios and more factors influencing consumer behaviors. In this context, the study needs to use a large amount of data to able to verify the differences in the effects produced by different advertising images. Second, the effectiveness metrics are different. Experiments measure advertising effectiveness in terms of advertising attitudes, product attitudes, and purchase intentions. In reality, however, there is not always a consistent relationship between consumer intentions and behaviors. In contrast, this paper extends the measurement from consumer intention to actual purchase by using real advertising campaign data. The data used in this study include not only the ad clicks that represent consumer intentions but also the conversions that represent the actual purchase. The inconsistency of the trends in CTR and CVR can indicate a discrepancy between consumer intention and behavior. In addition, this paper uses the construal level theory to accomplish the improvement of the efficiency of computational advertising, which not only contributes to psychological theory but also provides an actionable method for the iteration of the computational advertising system. Examples of previous studies are shown in Table 1 in comparison with this study.

**Table 1:** Study comparison with relevant literature.

Authors (Year)	Independent variable		Dependent variable		Theory	Data source	Key findings
	Dimension	Coding method	Evaluation	Action			
Park and Morton (2015)	Socially distance	Human	Attitudes toward advertising, intentions to action		Regulatory focus	Lab Experiments	Results indicate that when asked to make judgments for distant entities, individuals are more persuaded by a promotion-focused frame in terms of ad attitudes, whereas there are no differential framing effects on judgments associated with proximal entities.
Li and Xie (2020)	Image content and characteristics, Image-Text fit	Human and automatic	Attention(Inferred) and Engagement(Measured)		N.A	Field Data	The authors find a significant and robust positive mere presence effect of image content on user engagement in both product categories on Twitter. High-quality and professionally shot pictures consistently lead to higher engagement on both platforms for both product categories.
Jia et al. (2017)	Physical distance from the verbal description	Human	Beliefs		Mental image	Lab Experiments	Consumers’ physical distance from the verbal description of an event or a product can influence their beliefs in its implications. These and other effects are mediated by the vividness of the mental image.
Kim et al. (2019b)	Image proximity, product category	Human	Attitudes toward the ad, attitudes toward the product, purchase intentions		Construal level theory	Lab Experiments	Utilitarian products will cause low-level construal to match more strongly with rational appeals; hedonic products will cause high-level construal to match more strongly with emotional appeals.
Wang and Lehto (2020)	Congruency of color and message type	Human	Attitude toward the ad, attitude toward the restaurant, purchase intention, willingness to pay		Construal level theory	Lab Experiments	Taste-focused advertising messages combined with color imagery and health-focused advertising messages combined with black-and-white (BW) imagery can effectively boost consumer responses, including attitude toward the ad, attitude toward the restaurant, purchase intention, and willingness to pay (WTP).
Pieters et al. (2010)	Feature complexity, design complexity	Human	attention to the brand, attention to the advertisement, attitude toward the ad		Visual complexity theory	Lab experiments	Feature complexity hurts attention to the brand and attitude toward the ad, whereas design complexity helps attention to both the pictorial and the ad as a whole, its comprehensibility, and attitude toward the ad.
Henderson et al. (2019)	Sponsor–team visual congruence	Human	Brand recall, brand attitude, visit intentions, and purchase intentions		Attribution theory	Field data	Two experiments in the contexts of product packaging and online advertising provide converging evidence of the positive effects of created visual congruence on attitudes toward sponsorship, brand attitudes, and intentions.
Roose et al. (2019)	Consistency of visual and verbal elements	Human	Ad attitude, attractiveness, purchase intention		Construal level theory	Lab experiments	Advertising effectiveness increases when visual advertising elements (e.g., view height) and verbal advertising elements (e.g., time effectiveness) induce the same construal level.
Current study	Visual proximity in images	Human and automatic	CTR	CVR	Construal level theory	Field data	The results show that visually distant images can be more attractive, that is, higher CTR; visually proximate images can be more persuasive, that is, higher CVR.

### Two metrics in the sales funnel: CTR and CVR

The goal of computational advertising is to achieve a more efficient allocation of advertising resources through better targeting and to increase efficiency through enhanced ad relevance and personalization (Huh and Malthouse, 2020). Therefore, computational advertising updates algorithms parameters by continuously learning about ad effectiveness metrics to achieve optimized results. Among the commonly used ad effectiveness metrics, CTR and CVR are particularly valued (Helberger et al., 2020; Yun et al., 2020). The online advertising market is two-sided, and the interests of the media platforms and advertisers are not the same. The media platforms earn revenue by selling internet traffic, and therefore value CTR as the quality standard of internet traffic; the advertisers, as the buyers of internet traffic, not only care about CTR, but also the actual advertising revenue, namely, the number of conversions or purchases, and therefore pay more attention to the CVR that the ads can obtain(Choi et al., 2020). Different metric weights can influence the optimization direction of the computational advertising system, and therefore need to be discussed separately. In this study, we analyze the association between consumers’ cognitive states and their decision-making journey on the consumer level by using the construal level theory.

Following the previous studies on the consumer decision journey, this study divides the consumer decision journey into three stages: pre-purchase, purchase, and post-purchase (Hamilton and Price, 2019; Humphreys et al., 2021). The consumer’s decision journey, which is also referred to as the sales funnel, includes different segmentation methods such as identification, initial consideration, positive evaluation, purchase, and post-purchase behavior (Kietzmann et al., 2018). In the context of computational advertising, the system decides whether to engage in ad bidding for display opportunities based on consumer tags and ad scenarios. When the bidding is successful, the advertiser sends the ad material to the media platform, the media platform completes the ad display, the consumer receives the ad message, and the system records one impression. If the ad arouses consumers’ interest, consumers will click on it and complete the first step of the decision-making journey, and the system records it as one click and uses CTR to reflect this attractiveness. And if the ad persuades consumers to convert, consumers will make more time-consuming and laborious operations such as purchase or download, at which time consumers complete the second step of the decision-making journey, the system records it as one purchase or conversion and uses CVR to reflect this persuasiveness (Humphreys et al., 2021). The whole process is automated by the computational advertising system and is automatically fed back to the model for parameter iteration and optimization.

This study studies the impact of ad images on consumers’ evaluating, clicking, and conversion behavior in computational advertising, so this study chooses CTR and CVR to represent the probability of action in the first two stages of the consumer decision journey, respectively (Wang et al., 2019). What is clear is that clicks and conversions do not always occur, and there is a huge gap between product evaluation and actual purchase decisions in the consumer’s decision journey (Kietzmann et al., 2018; Humphreys et al., 2021). Even if consumers have the intention to buy, it remains uncertain whether they are willing to invest money or time resources to make the purchase, and the personalized adaptation of advertising content can play an important role in guiding consumers’ decision-making process. In this paper, we use construal level theory to analyze consumers’ cognitive states and use this mechanism to analyze consumer behavior and select advertising content for construal level matching to achieve the purpose of improving advertising effectiveness.

### Hypothesis development

Using the construal level theory framework, the consistency of matching image design has been explored in previous studies (Amit et al., 2009; Aydin, 2018), for example, black and white photos evoking high construal levels with health targets work better, and color photos evoking low construal levels with flavorful ads (Wang and Lehto, 2020). Matching congruence is defined as a good fit between things that may stem from conceptual or emotional similarities, such as objects, goals, ways of achieving them, related uses, and emotions or feelings (Maille and Fleck, 2011). The two core concepts in this study share conceptual similarities and are connected through the construal level theoretical framework, which promotes coherence.

The matching consistency of various elements in ads is an effective way to enhance persuasiveness (Fleck et al., 2012; Segev et al., 2014). In other words, persuasion is effective when the content of the ad matches the consumer’s cognitive, motivational, or emotional state (Cesario et al., 2004). For example, thematic congruence between magazines and ads can lead to more positive ad attitudes (Moorman et al., 2002). This matching consistency usually leads to positive outcomes (Camacho et al., 2003; Lee and Aaker, 2004), and in this study, matching the ad images with the consumer’s decision-making stage by the construal level theory can play a propulsive role in driving consumer action (Lee et al., 2010; Kareklas et al., 2019), leading to better advertising effectiveness.

As mentioned above, as the consumer decision journey advances, the psychological distance to the purchase goal gets closer. The goal of the pre-purchase stage is to gather information and expand the choice set without rushing to make a decision; in the purchase or conversion stage, the consumer’s goal changes to focusing on the product and making the purchase decision (Hamilton and Price, 2019). Thus, consumers’ thought patterns tend to be more abstract, i.e., at higher construal levels, in the pre-purchase stage; in the latter stages of the decision journey, consumers are closer to the actual conversion or purchase operation and their thought patterns are more focused, i.e., lower construal levels (Humphreys et al., 2021). Visually distant images evoke a distant psychological distance or high construal level, consistent with the pre-purchase stage, i.e., CTR, while visually proximate images evoke a near psychological distance or low construal level, consistent with the later decision journey, i.e., CVR. Specifically reflected in the advertising effectiveness metrics, the following assumptions can be made.

*H1:* Compared to visually proximate images, visually distant images are more effective for getting a higher CTR.
*H2:* Compared to visually distant images, visually proximate images are more effective for getting a higher CVR.

## Materials and methods

### Data

The data in this study came from a large Chinese game company that operates different types of games, and we use ad campaign data for one of the new games. The ad campaign for this new game relied on digital channels, with ads displayed on social media platforms to drive consumers to the game’s download page or app store and convince them to download it. The advertising campaign started on February 2, 2018, and lasted 168 days. In this paper, the results of the ad campaign on Weibo, which is a social media platform, are selected to test the hypothesis. The ads on it included 116 different ad images and 249 different combinations of ad text and images. This data are generated from 249 ad combinations of placements, where the data generated by a single ad in a day are recorded as a single row. The row includes ad text, ad image, ad display time, ad impressions, ad cost, ad clicks, and conversions. Each ad is allowed to run for several days, depending on whether the ad achieves satisfactory results for the firm. If the ad is not effective enough on the first day, the firm will not continue to choose the ad for the second day. At the end of 168 days of the advertising campaign, the campaign generated a total of 12,948 lines of data. The above description of advertising cost and effectiveness is shown in the Table 2.

**Table 2:** Statistics of the dataset.

	Min	Max	Mean	Std
Money	0.01	13733.86	110.22	443.31
Impressions	1	1,181,816	9051.34	36042.30
Clicks	0	31,740	108.69	657.11
Conversions	0	292	1.38	8.13
CTR	0.000	1.000	0.010	0.02
CVR	0.000	1.000	0.009	0.04

After completing the basic statistics of the data, this study also faces two problems: (1) the sparsity of the data is high, because the average value of both CTR and CVR is less than 1%, and many ads do not even generate clicks and conversions when they are placed, so the CTR and CVR are both 0, thus causing such a severe sparsity problem; (2) the endogeneity problem, i.e., firms decide whether to continue to deliver ads based on the effect of the previous day’s ads, so at the end of the 168-day-long campaign, the ad data retained in the dataset are the data generated by the selected ads, thus causing the endogeneity problem of self-selection bias.

For the sparsity problem, this paper uses two approaches to solve it: first, many zero values are eliminated from the dataset and cannot interfere with the normal data; then, the Tobit model is chosen to complete the correction of the non-zero data, and the results of the two models are compared with each other using OLS as the benchmark to complete the robustness test.

**Figure 1:**
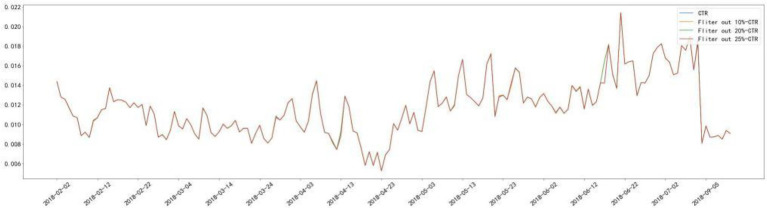
CTR comparison.

**Figure 2:**
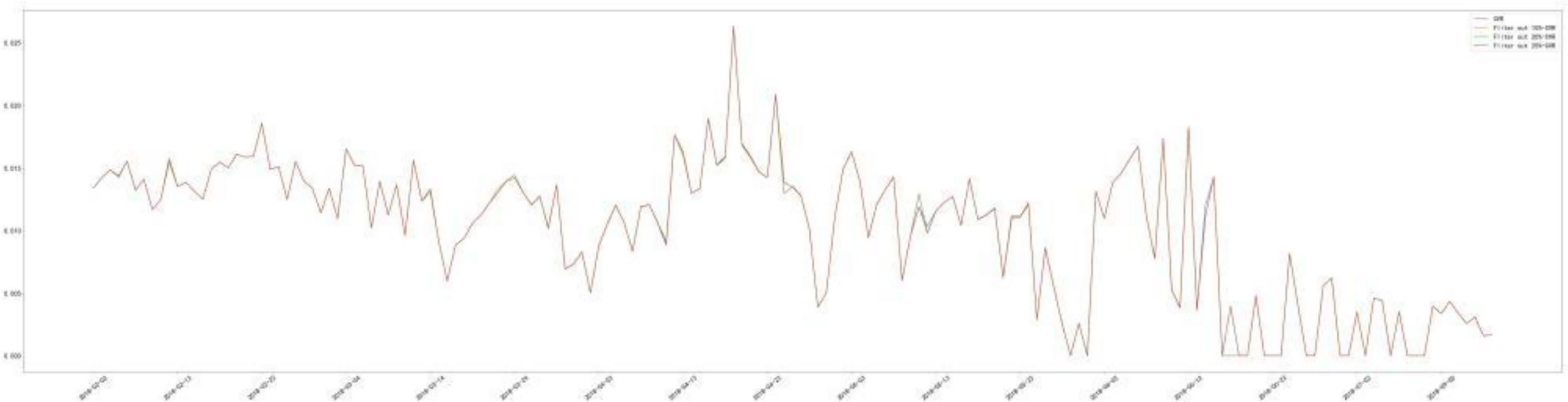
CVR comparison.

In this paper, we first select the zero values in the dataset, because sparsity arises from the fact that the ad cannot produce clicks and conversions, and once the ad is invested and displayed a small enough number of times, then it can be concluded that the ad will fail to produce any effect. Therefore, we conducted the statistics on the quantiles of the ad costs and chose to exclude the data of the last 25% of the ad costs, i.e., the data of the day when the ad costs were less than 0.685 yuan. It can be argued that when the money invested in advertising for a day is less than 0.685 yuan, the ads are not enough to generate sufficient impressions and lead to zero clicks and conversions, thus leading to an increase in data sparsity. In addition for the rest of the data, the daily CTR and CVR in the data set are almost unaffected after excluding this part of the data, which is compared in Figures 1, 2.

After cleaning the dataset, there are still 8,594 rows of data in the data, which contains 119,519,545 ad impressions, with a total ad spend of 1465460.2 yuan, generating a total of 1,358,964 ad clicks and 18,321 conversions. The range of ad campaigns covers a sufficient number of displays to a sufficient number of people to be considered that different demographic characteristics are covered in the data to accurately analyze and measure the effectiveness of the ads (Lewis et al., 2011; Lewis and Reiley, 2014). The data set variable correlations are shown in Table 3.

**Table 3:** Correlations of the variables.

	CTR	CVR	Money	Impressions	Image	Format	Text	Emotion	Days	Holiday	Texture	Color	Bright
CTR	1												
CVR	−0.045^**^	1											
Money	0.048^**^	0.036^**^	1										
Impressions	0.047^**^	0.019	0.948^**^	1					*				
Image	0.059^**^	−0.039^**^	−0.058^**^	−0.037^**^	1								
Format	0.108^**^	−0.090^**^	−0.149^**^	−0.152^**^	−0.008	1							
Text	0.058^**^	−0.037^**^	0.066^**^	0.058^**^	−0.118^**^	−0.011	1						
Emotion	−0.113^**^	0.008	−0.138^**^	−0.114^**^	0.107^**^	0.015	−0.166^**^	1					
Days	0.093^**^	−0.092^**^	−0.231^**^	−0.251^**^	−0.007	0.390^**^	0.093^**^	−0.057^**^	1				
Holiday	−0.049^**^	−0.012	−0.024^*^	−0.024^*^	0.008	0.013	0.009	−0.004	0.064^**^	1			
Texture	−0.044^**^	0.043^**^	−0.001	−0.011	0.066^**^	−0.257^**^	−0.015	−0.011	−0.128^**^	0.000	1		
Color	−0.088^**^	−0.001	0.006	0.010	−0.242^**^	0.000	0.105^**^	−0.016	−0.051^**^	−0.003	0.023^*^	1	
Bright	0.022^*^	−0.054^**^	−0.006	−0.019	0.051^**^	0.287^**^	0.262^**^	−0.061^**^	0.107^**^	0.006	−0.110^**^	0.251^**^	1

*p < 0.05, **p < 0.01.

### Machine learning-based variables coding

**Figure 3:**
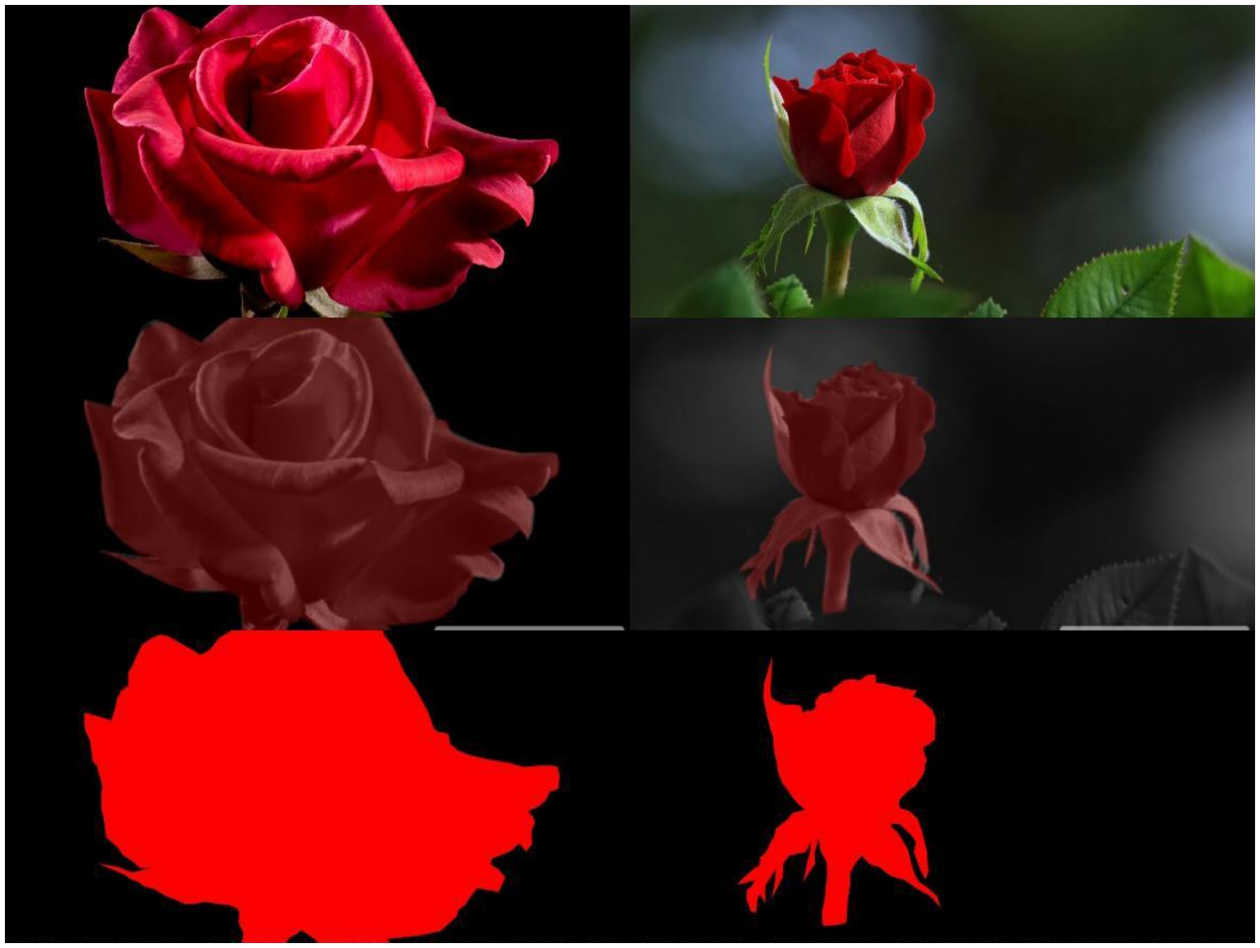
Examples of visually distant and proximate images.

Before applying the econometric model to analyze the data, we first need to classify the visually distant and proximate images. In this paper, we use a combination of machine learning and manual methods to complete the data coding. Firstly, the machine learning part of this paper uses a criterion of framing, i.e., the proportion of the frame occupied by the subject of the image. In this paper, we use OpenCV to extract the subject and background of the picture and calculate the proportion of the subject in the foreground in the picture to complete the first step of classification, and the examples are in Figure 3.

In the second step of manual coding, we recruited two separate groups, including 10 adult students from business schools who had already developed the concept of framing and visual distance, to participate in this work. Since the first step solved part of the problem, the algorithm still had difficulty determining the difference between the foreground and background. Therefore, we built on the results from the first step by manually coding whether the subject was foreground. We used a seven-level Likert scale (1 = near, 7 = far) to classify this. After scoring, we set thresholds for binary classification (1–4 for near and 4–7 for far) after taking the mean value of the scores of individual images to complete the binary classification of visually distant and proximate images. The agreement among members was high (γ = 0.90), and finally the disagreement was resolved by discussion.

**Figure 4:**
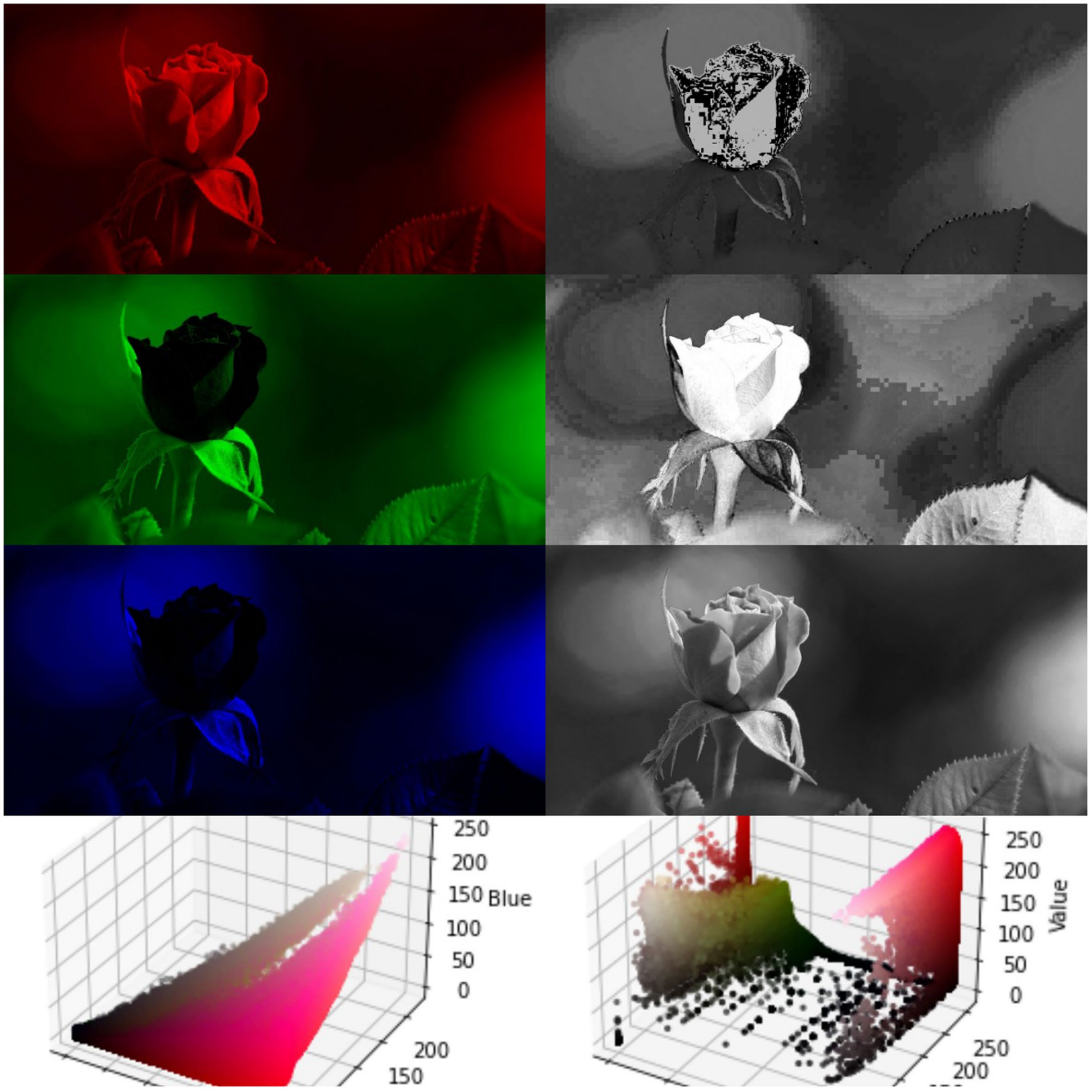
Comparison of RGB and HSV decomposition.

**Figure 5:**
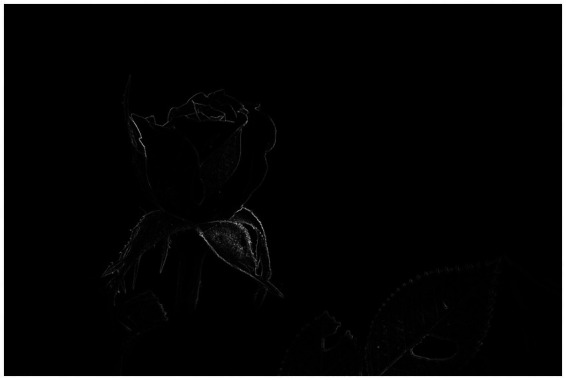
Example of extracting image texture.

Among the 116 different advertisement images in the result data, there were 51 visually proximate images, accounting for 43.97%, and 65 visually distant images, accounting for 56.03%. In addition to the content of the images, the feature variables of the images themselves are also added to the model in this paper. The feature variables of the images themselves include image texture (Pieters et al., 2010), image color, and lightness and darkness (Zhou et al., 2021). The texture part is extracted using the Sobel operator in OpenCV, and then the top five feature values are averaged by applying the PCA model (Li and Xie, 2020). The image color and brightness are calculated using Pillow in Python, and the RGB or HSV decomposition is performed and averaged according to the requirements. Since the size of the ad images on the Weibo platform needs to be standardized, the size and pixels of the images in this data are kept equal and do not need to be controlled separately. An example of RGB decomposition and HSV decomposition is shown in Figures 4, 5.

Beside ad images, the data also include the number of ad impressions, ad campaign time, ad text, daily ad cost, ad clicks, and conversions. In this study, we coded ad text according to previous literature, using speech act theory to classify ad copy into two categories, i.e., subjective text and objective text (Huang and Liu, 2022). Subjective texts focused on the personalized text providing joy or experience, which would lead to better ad attitude and product evaluation, and objective texts focused on the brand information trying to convince consumers to buy which would lead to better brand attitude and persuasiveness (Chen and Qasim, 2021; Huang and Liu, 2022). Even though this paper focuses on the influence of advertising images on consumer behavior, different types of advertising text can evoke differences in consumers’ construal levels, and thus can be used to justify the theoretical mechanism of this study from the side. In addition, since the emotion in ad text can influence consumer behaviors (Ordenes et al., 2018; ShiYong et al., 2021), this paper also adds the emotion of text as a control variable. To avoid artificial factors that affect the classification results, we use the API provided by Baidu PaddlePaddle to estimate the positive and negative sentiment in the ad text and obtain continuous variables.

### Heckman model

As mentioned earlier, this paper faces two major challenges in choosing the model: data sparsity and endogeneity. In the next part, this paper will use the Tobit model to solve the sparsity problem (left truncated tail) in many leads to get more stable results and use OLS as a benchmark for comparison to verify the robustness of the model results. In the face of endogeneity, the Heckman two-stage model is chosen to correct self-selection bias in the data by adding instrumental variables. Self-selection bias is also known as “essential heterogeneity,” which means that there is a correlation between endogenous variables and their effects. Essential heterogeneity leads to the problem of assessing empirical models and how effects are distributed across the population. Under essential heterogeneity conditions, the classical 2SLS model is no longer applicable and needs to be modified using the Heckman model (Heckman, 1979; Sande and Ghosh, 2018).

Specifically for this study, the self-selection bias arises from the fact that firms do not place all of their ads every day, but rather choose to deliver ads that work well. Firms tend to choose ads that are more effective rather than continuing to invest in less effective ads, e.g., firms decide whether to continue on the second day based on the performance of the ads on the first day. Therefore, we use a two-stage Heckman model to address the endogeneity of the ad campaign. We add a new variable “continuity” to the model to represent the continuous choice of ad delivery. The value of this variable is set to 1 when the ad is delivered on the second day and 0 when the ad is not displayed on the second day, as shown in Table 4 below.

**Table 4:** Constructs and measures.

Variable’s name (name in the equation)	Variable description
**Dependent variable**	
CTR	CTR = clicks/exposures
CVR	CVR = conversions/clicks
Independent variables	
Image	Machine learning mixed with manual coding, the visually proximate image is recorded as 0; the visually distant image is recorded as 1.
Format	The design of the ad, banner ad as 0; native ad as 1.
Text	Machine learning mixed with manual coding, the objective text is recorded as 0, and the subjective text is recorded as 1
**Control variables**	
Emotion	The emotion of the ad text is calculated by Baidu PaddlePaddle.
Days	The number of days since this ad is exposed.
Workdays	The day is recorded as 0 when it is a holiday; the day is recorded as 1 when it is a workday.
Texture	Extracted by OpenCV, and PCA model was used to calculate the first five feature values and then average them for the final number.
Color	The color richness of images, continuous variable, and the arithmetic average of the color of pictures in RGB space.
Brightness	Illumination elements in an image, continuous variables, using Pillow to calculate the root mean square value of pixel value of each channel in HSV space.
**Instrumental variables**	
Continuity	1, when the ad is launched on the following day; 0, when the ad is not launched on the following day.
Money	The money cost by this ad in this day.
Impressions	The impressions of this ad on this day

In the first stage of the Heckman model, we regressed continuity on all independent and instrumental variables to obtain IMR. For these firm-level variables, we unified the timing of all independent variables on the same day. In addition, we include advertising effectiveness metrics (CTR, CVR) in the regressions to correct for self-selection bias. Due to Weibo’s regulations on ad formats, the sizes of images in the data are all equal and do not need to be controlled separately. We use ad cost and impressions as instrumental variables because consumers have no way of knowing the cost of an ad or the number of times an ad is displayed on the platform when they view it, and consumer behavior is not affected by either variable. However, the two variables affect the firm’s delivery choice, and when the number of impressions and cost appear to be too high compared to the effectiveness of the ad, the firm will no longer make a delivery decision, so the instrumental variables are chosen as required. In the first stage of the model, the Inverse Mills Ratio (IMR) can be derived and is embedded in the second stage to correct for self-selection bias.

In the second stage of the Heckman model, we add IMR to the regression model in that stage while removing the instrumental variables from the first stage. In addition to this, the Tobit model was used in this study to address the presence of a large number of zeros in the data. The results of the regressions remain stable and the interactions reveal more information about the advertising design. The IMR coefficients explain and correct the self-selection bias affecting the outcome variables. Our results show that the IMR coefficient is negative and significant (*p* < 0.001), indicating that the selection correction term adjusts downward for self-selection bias. Due to the specific data structure, we use a log transformation to bring the data closer to normal distribution. The specific Heckman model equation can be written as:


$$\mathrm{Prob}\left(\mathrm{continuity}=1\right)=\alpha_{0}+\alpha_{11}\mathrm{Money}+\alpha_{12}\mathrm{Impressions}+\beta_{11}$$

$$\mathrm{Images}+\beta_{12}\mathrm{Format}+\beta_{13}\mathrm{Text}\gamma_{11}\mathrm{Emotion+}\gamma_{12}\mathrm{Holiday}$$

$$\begin{array}{l} \gamma_{13}\mathrm{Days}+\gamma_{14}\mathrm{Texture}+\gamma_{15}\mathrm{Color}+\gamma_{16}\mathrm{Brightness}+\mu {CTR}_{n}^{\prime }\\ \left(\mathrm{or}\;{CVR}_{n}^{\prime }\right) \end{array}$$

where Money and Impressions are the two instrumental variables. ${CTR}_{n}^{\prime }$ =ln(100* $CTR_{n}$ +1) and ${CVR}_{n}^{\prime }$ =ln(100* $CVR_{n}$ +1) represents the results of the ad campaign on day n.The second stage of Heckman model can be written as:

)$$\begin{array}{l} {CTR}_{n+1}^{\prime }\left(\mathrm{or}\;{CVR}_{n+1}^{\prime }\right)=f_{1}(\beta_{0}+\beta_{21}\mathrm{Image}\\ +\beta_{22}\mathrm{Format}+\beta_{23}\mathrm{Text}+\gamma_{21}\mathrm{Emotion}\\ +\gamma_{22}\mathrm{Holiday}+\gamma_{23}\mathrm{Days}+\gamma_{24}\mathrm{Texture}\\ +\gamma_{25}\mathrm{Color}+\gamma_{26}\mathrm{Brightness}+{IMR}_{CTR}\\ \left(\mathrm{or}\;{IMR}_{CVR}\right) \end{array}$$

The interaction part is:


$${CTR}_{n+1}^{\prime }\left(\mathrm{or}\;{CVR}_{n+1}^{\prime }\right)=f_{1}\left(\begin{array}{l} \beta_{0}+\beta_{31}\mathrm{Image}+\beta_{32}\mathrm{Format}\\ +\beta_{33}\mathrm{Text}+\rho_{31}\mathrm{Image}\\ {}*\mathrm{Format}+\rho_{32}\mathrm{Image}\\ {}*\mathrm{Text}+\rho_{33}\mathrm{Format}*\mathrm{Text}\\ +\gamma_{31}\mathrm{Emotion}+\gamma_{32}\mathrm{Holiday}\\ +\gamma_{33}\mathrm{Days}+\gamma_{34}\mathrm{Texture}\\ +\gamma_{35}\mathrm{Color}+\gamma_{36}\mathrm{Brightness}\\ +{IMR}_{CTR}\left(\mathrm{or}\;{IMR}_{CVR}\right) \end{array}\right)$$

where ${CTR}_{n+1}^{\prime }$ =ln(100* ${CTR}_{n+1}$ +1) and ${CVR}_{n+1}^{\prime }$ =ln(100* ${CVR}_{n+1}$ +1) represent CTR and CVR on the (*n* + 1)th day, ${IMR}_{CTR}，{IMR}_{CVR}$ are the inverse Mills ratios from the first stage of the two models, respectively.

The main effect outcomes are shown below in the Table 5.

**Table 5:** Main effects results.

	OLS		Tobit	
	CTR	CVR	CTR	CVR
Images	0.078^***^	−0.075^***^	0.085^***^	−0.123^*^
Format	0.052^***^	−0.437^***^	0.012	−2.411^***^
Text	0.053^***^	−0.123^***^	0.050^***^	−0.386^***^
Emotion	−0.211^***^	−0.057^**^	−0.236^***^	−0.397^***^
Holiday	−0.020^*^	0.077^***^	−0.012	0.304^***^
Days	0.001^***^	−0.001	0.001^***^	−0.002^*^
Texture	−2.322^***^	−4.463^***^	−2.748^***^	−18.062^***^
Color	−0.003^***^	−0.001	−0.004^***^	−0.001
Brightness	−0.001^***^	0.001	−0.001^***^	−0.001
IMR	−0.665^***^	−3.883^***^	−1.162^***^	−18.002^***^

*p* < 0.10.

* *p* < 0.05;

** *p* < 0.01;

*** *p* < 0.001.

After estimating the main effects, both H1 and H2 are verified. Visually distant images get higher CTR ( $OLS:\beta_{21}^{CTR}=0.078$ , *p* < 0.001; $\mathrm{Tobit}:\beta_{21}^{CTR}=0.085\text{,}$ *p* < 0.001), visually proximate images get higher CVR (OLS: $\beta_{21}^{CVR}=-0.075$ , *p* < 0.001; Tobit: $\beta_{21}^{CVR}=-0.123$ , *p* < 0.05). In addition, differences in ad formats can affect the effectiveness of ads to varying degrees, with native ads having higher CTR ( $OLS:\beta_{22}^{CTR}=0.052$ , *p* < 0.001; $\mathrm{Tobit}:\beta_{22}^{CTR}=0.012$ , N.S); in contrast, banner advertising has not shown many advantages in previous studies, but in the context of computational advertising, banner advertising leads to higher CVR ( $OLS:\beta_{22}^{CVR}=-0.437$ , *p* < 0.001; $OLS:\beta_{22}^{CVR}=-2.411$ , *p* < 0.001). In terms of advertising text, we can still verify this paper’s theoretical mechanism. The subjective text emphasizes the pleasure and experience that the ad and the product can bring to the consumer, emphasizing the attractiveness of the ad, and the consumer is evoked with an abstract way of processing information and a higher construal level that can lead to a higher CTR ( $OLS:\beta_{23}^{CTR}=0.053$ , *p* < 0.001; $OLS:\beta_{23}^{CTR}=0.050$ , *p* < 0.001); objective text emphasizes brand characteristics and efficacy, emphasizing the persuasive power of the brand in the ad, and consumers are evoked with figurative information processing and a low construal level, which can lead to higher CVR ( $OLS:\beta_{23}^{CVR}=-0.123$ , *p* < 0.001; $OLS:\beta_{23}^{CVR}=-0.386$ , *p* < 0.001), the correspondence between the design of advertising text and advertising effectiveness can be a side note to the correctness of the construal level theory matching mechanism used in this paper. Next, we will analyze the results of the model interaction terms, the results are shown in Table 6.

**Table 6:** Interaction results.

	OLS		Tobit	
	CTR	CVR	CTR	CVR
Images	0.033^*^	−0.077^**^	0.028^*^	−0.091.
Format	0.068^**^	−0.515^***^	0.023	−2.516^***^
Text	0.033^*^	−0.137^***^	0.025	−0.386^***^
Images*Format	0.042^*^	0.041	0.067^**^	−0.084
Images*Text	0.068^***^	−0.023	0.080^***^	−0.049
Format*Text	−0.072^***^	0.121^***^	−0.086^***^	0.293
Emotion	−0.202^***^	−0.073^***^	−0.225^***^	−0.414^***^
Holiday	−0.020^*^	0.066^***^	−0.012	0.304^***^
Days	0.001^***^	−0.001	0.001^***^	−0.002^*^
Textual	−1.968^***^	−4.592^***^	−2.305^***^	−18.361^***^
Color	−0.003^***^	−0.001	−0.003^***^	−0.001
Brightness	−0.001^***^	0.001	−0.001^***^	−0.001
IMR	−0.668^***^	−3.482^***^	−1.163^***^	−17.983^***^

*p* < 0.10.

* *p* < 0.05;

** *p* < 0.01;

*** *p* < 0.001.

After adding the interaction term, H1 and H2 are again verified. Visually distant images get higher CTR (OLS: $\beta_{31}^{CTR}=0.033$ , *p* < 0.05; Tobit: $\beta_{31}^{CTR}=0.028$ , *p* < 0.05), visually proximate images get higher CVR ( $OLS:\beta_{31}^{CVR}=-0.077$ , *p* < 0.01; $\mathrm{Tobit}:\beta_{31}^{CVR}=-0.091$ , *p* < 0.1). In terms of ad formats, native ads still have an advantage in terms of CTR (OLS: $\beta_{32}^{CTR}=0.068$ , *p* < 0.05; Tobit: $\beta_{32}^{CTR}=0.023$ , N.S.); banner ads perform better in terms of CVR ( $OLS:\beta_{32}^{CVR}=-0.515$ , *p* < 0.001; $\mathrm{Tobit}:\beta_{32}^{CVR}=-2.516$ , *p* < 0.001). The choice of ad text also validates the effect of construal level matching, with subjective text leading to higher CTR (OLS: $\beta_{33}^{CTR}=0.033$ , *p* < 0.05; Tobit: $\beta_{31}^{CTR}=0.025$ , N.S.) and the objective text leads to higher CVR ( $OLS:\beta_{33}^{CVR}=-0.137$ , *p* < 0.001; $\mathrm{Tobit}:\beta_{33}^{CVR}=-0.386$ , *p* < 0.001). In terms of interaction, native ads with visually distant images can lead to higher CTR (OLS: $\rho_{31}^{CTR}=0.042$ , *p* < 0.05; Tobit: $\rho_{31}^{CTR}=0.067$ , *p* < 0.01), but it does not show the advantage of matching in terms of CVR (OLS: $\rho_{31}^{CVR}=0.041$ , N.S.; Tobit: $\rho_{31}^{CVR}=-0.084$ , N.S.). What is more important is the method of matching ad text with images. According to the principle of construal level matching, subjective text with visually distant pictures can bring an increase in CTR (OLS: $\rho_{32}^{CTR}=0.068$ , *p* < 0.001; Tobit: $\rho_{32}^{CTR}=0.080$ , *p* < 0.001), objective text with visually proximate images does not lead to higher CVR (OLS: $\rho_{32}^{CVR}=-0.023$ , N.S.; Tobit: $\rho_{32}^{CVR}=-0.049$ , N.S.). This suggests that consumers in the later stage of the decision journey can allocate more cognitive resources to measuring the gains and losses, resulting in significantly less salience in terms of CVR than CTR. The CVR result illustrates that in the context of computational advertising, where precise targeting and personalized content delivery are accomplished through data and algorithms, all are designed to allow consumers to make decisions in a very short time window. However, when consumers reach the purchase or conversion stage, the advancement of the decision journey allows consumers to invest more resources in measuring the gains and losses, making the CVR not as sensitive as the CTR. The analysis of the model also illustrates the theoretical and practical value provided by this study in guiding the design of ads, with specific contributions described below.

## General discussion

### Theoretical contribution

With its personalization and automation, computational advertising is becoming increasingly popular (Choi et al., 2020). Firms use artificial intelligence models to match content based on personalized consumer tags to improve the effectiveness of computational advertising (Chen et al., 2019; Deng et al., 2019). However, current data-driven computational advertising faces problems, such as low computational efficiency, over-reliance on the correlation between data and little consideration of causality between data when performing the above steps, lack of content generation guidelines, and unclear iterative directions. This study adds causal inference capability to the advertising system by introducing the construal level theory to match advertising content with consumer psychological states in the same theoretical framework, which helps to understand the design method of ad images and improve advertising effectiveness (Hernandez et al., 2015). In terms of ad effectiveness metrics, this study uses CTR and CVR as effectiveness metrics, where CTR can measure the attractiveness of an ad and CVR can measure the persuasiveness of an ad (Aribarg and Schwartz, 2020; Choi et al., 2020), with CTR representing the pre-purchase stage of consumer decision journey and CVR representing the purchase stage of consumer decision journey (Humphreys et al., 2021). Within the framework of construal level theory, this study relates the two effectiveness metrics to image design, with visually distant images better matching the construal level of the pre-purchase stage and visually proximate images better matching the construal level of the purchase stage. Reflecting on the effectiveness metrics, the ads achieve better CTR when using visually distant images and better CVR when using visually proximate images. The theoretical contributions of this paper are as follows:

1. In this paper, we use the construal level theory to construct the guidelines for the design of advertising images in the context of computational advertising. The contributions are mainly in two aspects: first, current computational advertising relies on data and models, ignoring the changes in consumers’ psychological states, which makes it difficult to generalize advertising design methods and wastes advertising resources. This paper, however, uses construal level theory to connect ad content with consumer behavior, suggesting new guidelines for image design and indicating iterative directions to better move toward personalization goals; second, previous studies of advertising images have focused on traditional forms of advertising (Zhang et al., 2020), while computational advertising contexts in which consumers are in different states of information processing under traditional advertising conditions (Aribarg and Schwartz, 2020), it is difficult for the laboratory experiments to capture and imitate the consumption scenario. This paper then verifies the validity of the theoretical mechanism in complex consumption scenarios by examining the effects of advertising images on consumers’ psychological states in more complex contexts.
2. In this paper, two metrics for measuring advertising effectiveness, i.e., CTR and CVR, are matched with advertising images. Previous studies have mainly focused on consumers’ attitudes toward ads, attitudes toward products, and purchase intentions (Wang et al., 2019; Choi et al., 2020). Moreover, the purchase behavior of consumers or the conversion probability between different decision stages has not been studied. In this paper, CTR and CVR are linked to the sales funnel, where CTR represents the attractiveness of ads to consumers and belongs to the pre-purchase stage, and CVR represents the conversion probability of ads to consumers and belongs to the purchase stage. Under the framework of construal level theory, this study matches the advertising effectiveness metrics with the images and finally concludes that ads get higher CTR when using visually distant images and higher CVR when using visually proximate images.

### Management contribution

In the digital era, consumer attention has become a scarce resource that firms must gain. Computational advertising achieves higher efficiency than traditional advertising through personalized ad design and delivery by filtering consumer tags, locating consumer states, predicting consumer needs, and designing corresponding ad content to cater to consumer needs (Huh and Malthouse, 2020; Yun et al., 2020). After understanding the relationship between consumer psychological states and behaviors, as well as completing the ad design, this study can provide better iterative ideas for different players in the online advertising market (Helberger et al., 2020). In the online advertising market, different players value different advertising effectiveness metrics. Media platforms valued CTR more because they needed to price internet traffic based on CTR. Advertisers focused more on CVR because of the need for actual purchase and revenue (Choi et al., 2020). Firms needed to choose different advertising content according to different advertising campaign goals. Under the framework of construal level theory, this study investigated the impact of different advertising image designs on advertising effectiveness by taking CTR and CVR as indicators and giving suggestions for advertising design and strategies. The details are as follows:

1. Media platforms want to use higher CTR to price internet traffic, and this paper can provide ideas for system iteration. The CTR represents the attractiveness of the ad to the consumer, and the media platform should require the use of visually distant images and limit the use of visually proximate images when generating the ad contents. The use of visually distant images evokes a higher construal level when the image matches the psychological state of the consumer when viewing the ad. Consumers can generate better attitudes and more clicks, and better CTR can be fed back to the computational advertising system, which automatically adjusts the parameters to be more favorable to CTR and generate revenue for the media platform.
2. Advertisers want to get more purchases or conversions to earn profits, so they pay more attention to CVR. CVR represents the ability of ads to persuade consumers and corresponds to the later stages of the consumer decision making journey when consumers are at a low construal level. Visually proximate images can evoke the low construal level and bring better CVR by matching the psychological state. Advertisers should be more precise in targeting consumers’ decision states, use more visually proximate images and limit the use of visually distant images to get better CVR so that they can get more revenue when the system iterates automatically.

### Limitations and future directions

This study analyzed the impact of advertising images on consumer behaviors on social media platforms and drew some conclusions, but there were still some problems worth studying in the future. First of all, this study explored the influence of different image design methods on advertising effectiveness but did not consider the effects caused by advertising texts (Ordenes et al., 2018; Ha et al., 2020). Previous studies showed that texts work better if they match with ad images. In the future, advertising texts and images should be both incorporated into the construal level theory framework. Second, the data set used was generated by a single product. In the future, more datasets from different types of products should be used for analysis and to test the validity of the above conclusions. For example, ads for luxury goods also emphasized the distance between consumers and brands, the images used many visually distant elements. Third, this study examined the ads on the Weibo platform. Due to the difference in recommendation algorithms and the characteristics of media platforms, the effectiveness of advertising campaigns should be very different. In the future, the differences in advertising effectiveness between different media platforms should be worth studying.

## Data availability statement

The raw data supporting the conclusions of this article will be made available by the authors, without undue reservation.

## Author contributions

TL conceived, designed the study, and wrote the manuscript. ZY contributed to the revise of the manuscript. All authors contributed to the article and approved the submitted version.

## Conflict of interest

The authors declare that the research was conducted in the absence of any commercial or financial relationships that could be construed as a potential conflict of interest.

## Publisher’s note

All claims expressed in this article are solely those of the authors and do not necessarily represent those of their affiliated organizations, or those of the publisher, the editors and the reviewers. Any product that may be evaluated in this article, or claim that may be made by its manufacturer, is not guaranteed or endorsed by the publisher.
